# Modeling the Human Kinetic Adjustment Factor for Inhaled Volatile Organic Chemicals: Whole Population Approach versus Distinct Subpopulation Approach

**DOI:** 10.1155/2012/404329

**Published:** 2012-03-07

**Authors:** M. Valcke, A. Nong, K. Krishnan

**Affiliations:** ^1^Département de Santé Environnementale et de Santé au Travail, Université de Montréal, Montreal, QC, Canada H3T 1A8; ^2^Institut National de Santé Publique du Québec, Montréal, QC, Canada H2P 1E2

## Abstract

The objective of this study was to evaluate the impact of whole- and sub-population-related variabilities on the determination of the human kinetic adjustment factor (HKAF) used in risk assessment of inhaled volatile organic chemicals (VOCs). Monte Carlo simulations were applied to a steady-state algorithm to generate population distributions for blood concentrations (CAss) and rates of metabolism (RAMs) for inhalation exposures to benzene (BZ) and 1,4-dioxane (1,4-D). The simulated population consisted of various proportions of adults, elderly, children, neonates and pregnant women as per the Canadian demography. Subgroup-specific input parameters were obtained from the literature and P3M software. Under the “whole population” approach, the HKAF was computed as the ratio of the entire population's upper percentile value (99th, 95th) of dose metrics to the median value in either the entire population or the adult population. Under the “distinct subpopulation” approach, the upper percentile values in each subpopulation were considered, and the greatest resulting HKAF was retained. CAss-based HKAFs that considered the Canadian demography varied between 1.2 (BZ) and 2.8 (1,4-D). The “distinct subpopulation” CAss-based HKAF varied between 1.6 (BZ) and 8.5 (1,4-D). RAM-based HKAFs always remained below 1.6. Overall, this study evaluated for the first time the impact of underlying assumptions with respect to the interindividual variability considered (whole population or each subpopulation taken separately) when determining the HKAF.

## 1. Introduction

An interindividual variability (or uncertainty) factor (IVF) of a default value of 10 is usually applied to the point of departure (POD) for deriving reference doses (RfDs) or reference concentrations (RfCs) for use in noncancer risk assessment [1–3]. As reviewed by Price et al. [4], the IVF has historically been defined as a factor required to protect the sensitive members of the population since the POD is generally determined for average healthy individuals. Actually, two models have been proposed to describe the IVF. Under the “sensitive population” model, the IVF is applied to correct for the possible failure of a critical study to include a sufficient number of members pertaining to distinct subpopulation exhibiting an increased sensitivity. Conversely, under the “finite sample size” model, the application of the IVF relates to the possibility that the retained POD may fail to identify the toxicity threshold in the overall population simply because of the finite size of the sample in which it was determined [4]. Thus, the IVF accounts for the overall biological variability in the human population.

In the last 20 years, the IVF has been divided into two constitutive components (toxicokinetic and toxicodynamic factors), equal to 3.16 each based on pharmaceutical data [5–7]. This subdivision can be used in the evaluation of the magnitude and adequacy of the IVF for specific chemicals, and its replacement when appropriate data are available, by quantifying chemical-specific adjustment factors (CSAFs) [8, 9]. Under this method, the CSAF for interindividual variability in toxicokinetics, also referred to as the human kinetic adjustment factor (HKAF), can be determined based on the population distributions of relevant pharmacokinetic parameters (e.g., half-life, maximal concentration). The HKAF is calculated as the ratio between the upper percentile value of a parameter (i.e., 95th) and its central tendency value (i.e., median) in the whole population or between an upper percentile value in a presumed susceptible subpopulation and the central tendency value in the general healthy population [8, 9].

Neither the historical definitions of IVF [4] nor the IPCS guidance document on CSAFs [8] clearly defines the “average healthy individual,” forming the point of comparison for the presumed sensitive subpopulations. Particularly, it is unclear as to whether this individual is the average healthy adult or the average healthy individual from the whole population (which includes both healthy adults and sensitive subpopulations). But presumably because the POD used to derive the RfD or RfC is generally determined in healthy adults (animal or human) [10], HKAF evaluations conducted using experimental data for drugs [11–13] or PBPK model simulation data for environmental toxicants [14–16] have relied on what can be called a “distinct subpopulation” approach. Thus, the experimental or simulated data in the presumed susceptible individuals (e.g., neonates, pregnant women, elderly, polymorphic individuals) have often been compared with the corresponding data in healthy adults.

Alternatively, HKAF can be quantified using a “whole population” approach as done recently by Mörk and Johanson [17]. In this study, HKAFs were calculated for inhaled acetone based on a simulated distribution of steady-state blood concentration in an entire population, including adults and various age-defined groups of children. The PBPK modeling results in the different subgroups were weighted according to demographic representation in Sweden. Excluding the endogenous production of acetone, an HKAF of 1.9 was obtained by dividing the 95th percentile value of the entire population by the median. In comparison, using the 95th percentile value of that same dose metric in 3-month-old babies as well as 10 and 15 yr old children resulted in HKAFs of 2, 2.4, and 1.7, respectively.

The hypothesis that the HKAF determined upon the “whole population” approach is quite different from the one determined based on the “distinct subpopulation” approach stems from the results of Mörk and Johanson [17]. This potential difference could be significant from a regulatory standpoint because it may not lead to comparable levels of protection for the different subgroups that compose the whole population. It is also not known whether the population composition and the chemical considered may impact this potential difference. Thus, the objective of the current study was to evaluate the magnitude and adequacy of the HKAFs determined by the “whole population” approach as compared to the “distinct subpopulation” approach. In effect, population distributions of internal dose metrics following chronic exposure to two chemicals exhibiting different clearance characteristics were used to compute the HKAF as

- the ratio of the upper percentile value in the entire population including adults and nonadults over the median in adults and in this entire population;
- the ratio of the upper percentile value in presumed susceptible subpopulation over the median in adults and in the entire population including adults and non-adults.

## 2. Methods

A physiologically based steady-state algorithm combined with Monte Carlo simulation software was used to generate population distributions of blood concentration (CAss) and rate of metabolism (RAM) for chronic inhalation exposure to two chemicals with contrasting systemic clearance characteristics. The population distributions were reconstructed based on different proportions of randomly selected adults, elderly, children, neonates, and PW, and they were used to compute HKAFs based on “whole population” and “distinct subpopulation” approaches.

### 2.1. Selection of Surrogate Chemicals and Their Specific Parameters

Two VOCs were chosen as surrogate chemicals because they exhibit contrasting systemic clearances based on their pulmonary clearance potential (different blood : air partition coefficient (*P*_*b*_)) and their hepatic clearance (different hepatic extraction ratios). Benzene was chosen as an extensively cleared chemical because of its high pulmonary clearance (low *P*_*b*_, 7.4) and high hepatic extraction ratio. Conversely, 1,4-dioxane was chosen as a poorly cleared chemical due to its low pulmonary clearance (*P*_*b*_ = 3650) and low hepatic extraction ratio. While benzene is a known substrate of CYP2E1 [18], for which extensive data on interindividual variability are available [19, 20], 1,4-dioxane was included in this study to facilitate the coverage of a range of physico/biochemical properties of potential substrates of CYP2E1 [21]. Chemical-specific parameters are indicated in Table 1 and were taken from the literature [10, 22, 23]. The choice of these two surrogate VOCs and associated kinetic parameters was undertaken to reflect the range of kinetic characteristics of hypothetical substances for evaluating the HKAF. As such, the present modeling study did not focus on any aspect of the risk assessment relating to these specific chemicals.

### 2.2. Use of a Biologically Based Steady-State Model for the Simulation of Continuous Inhalation Exposure in Different Subpopulations

The current study relies on the use of a steady-state model for inhalation exposures (e.g., [24–27]), because the current study aimed at reconstructing population distributions of internal dose metrics for continuous lifetime exposures. Briefly, the algorithm computes the arterial blood concentration at steady-state (CAss) from the alveolar ventilation rate (Qp), the concentration in air (Ci), and the hepatic (Ql × *E*_hep_) and pulmonary (Qp/*P*_*b*_) clearances [27]:

(1) $$\text{CAss}=\frac{\text{Qp}\times \text{Ci}}{\text{Ql}\times E_{\text{hep}}+\text{Qp}/P_{b}},$$

where  Ql is the liver blood flow, *P*_*b*_ is the blood : air partition coefficient, and *E*_hep_ is the hepatic extraction ratio of the chemical and is calculated from its intrinsic clearance (Clint) as follows:

(2) $$E_{\text{hep}}=\frac{\text{Clint}}{\text{Clint}+\text{Ql}}.$$

Also, the rate of metabolised parent compound per unit volume of liver (RAM) is calculated as

(3) $$\text{RAM}=\frac{{{\text{CA}}_{\text{ss}}\times \text{Ql}\times E}_{\text{hep}}}{\text{Vl}}.$$

As indicated in Table 2, Qp, Ql, and Vl were calculated for a given individual by applying equations derived from Price et al. [28] to the individual's body weight [16]. The input data are listed in Table 2 for each subpopulation considered [15, 16, 19, 20, 28, 29]. These include six age groups covering the lifespan (neonates (0*–*30 d), infants (1*–*12 mo), toddlers (1*–*3 yr), children/adolescents (4*–*17 yr), adults (18*–*64 yr), and elderly (65*–*90 yr)), as well as pregnant women (15*–*44 yr). Ql and Vl for pregnant women were actually calculated on the basis of the body weight for nonpregnant women, whereas the appropriate increase in alveolar ventilation rate at any time during pregnancy was accounted for when computing Qp [16].

### 2.3. Generation of Distributions of Internal Dose Metrics by Means of Monte Carlo Simulations

Constant inhalation exposure to a benzene concentration corresponding to 10× the RfC (Table 1) was simulated in each subpopulation. Given the lack of an RfC for 1,4-dioxane, and its approximately tenfold greater RfD compared to benzene [10], a concentration that was ten times greater than the benzene concentration was specified. Monte Carlo simulations were performed using the Crystal Ball software (Oracle, Redwood Shores, CA) to generate distributions of the various internal dose metrics (see below). The intrinsic clearance in (2) was corrected for a given individual in any subpopulation by adjusting the maximum rate of metabolism (*V*max⁡_ind_) using enzyme-specific catalytic turnover [14–16]. This was determined based on the *V*max⁡ in an adult of average body weight (BW_avg_ad_), as well as the (individual (ind)/average adult (avg_ad)) ratios of the liver volumes and CYP2E1 hepatic content (in pmol/mg of microsomal protein):

(4) $$\begin{array}{cc} & {V\max}_{\text{ind}}\\ & \;\;=\frac{V_{\max_{\text{c}}}\times {{\text{BW}}_{\text{avg}\_\text{ad}}}^{0.75}}{{\left[\text{CYP}2\text{E}1\right]}_{\text{avg}\_\text{ad}}\times {\text{Vl}}_{\text{avg}\_\text{ad}}}\times {\left[\text{CYP}2\text{E}1\right]}_{\text{ind}}\times {\text{Vl}}_{\text{ind}}. \end{array}$$

A constant hepatic microsomal protein concentration was assumed across the subpopulations as discussed in Valcke and Krishnan [16].

#### 2.3.1. Distributions in the “Whole Population” and Corresponding HKAFs

Distributions of the internal dose metrics were generated for a theoretical population of 100,000 people with the demographic characteristics of Canada [30]. Therefore, the number of iterations used in the Monte Carlo simulations for each subpopulation corresponded to the targeted number of individuals. This number was based on the demographic proportions of each subpopulation (Table 3). Because the number of individuals appeared relatively constant across census' age ranges of same duration, the number of individuals pertaining to an age range different than those defined in the census could easily be estimated. For example, the number of toddlers aged 1–3 was considered as 60% of the total individuals 0–4 yr old. Finally, the number of pregnant women was calculated based on the pregnancy rate of 104/1,000 from Ventura et al. [31] and on the number of women aged 15–44 yr from the census data. The dose metric values “generated” by the Monte Carlo simulations for each subpopulation were then merged into a single “Canadian population dataset” of 100,000 values. To calculate the HKAFs based on the “whole population” approach, the ratio of the upper percentile value of the dose metric in the entire Canadian population to its median value was computed. The percentage of each subpopulation that was protected by a “whole population” HKAF was determined by identifying the number of individuals in each subpopulation exhibiting an internal dose metric that was lower than the entire population's upper percentile value underlying the HKAF, that is, 95th or 99th.

#### 2.3.2. Distributions in Each “Distinct Subpopulation” and Corresponding HKAFs

Distributions of the internal dose metrics for 100,000 individuals of each subpopulation were generated and the chemical- and dose-metric-specific HKAFs were calculated based on the “distinct subpopulation” approach, that is, as the ratio of the upper percentile value (i.e., 95th or 99th) in each subpopulation to the median value in adults or the whole Canadian population (see above). Also, for a given dose metric, the greatest “distinct subpopulation-” based HKAF was multiplied by the median in the whole Canadian population (see above) to obtain a threshold dose metric value. This threshold corresponded to the percentile that was referred to for determining the proportion of individuals from the entire population that was covered by the greatest “distinct subpopulation” HKAF.

### 2.4. Evaluation of the Impact of Demography on the Computed HKAFs

Given that the HKAF values as computed herein rely on the distribution of internal dose metrics in a general population composed of various proportions of each subpopulation, it was hypothesized that the demographic characteristics of a given general population may impact this calculation. To test this hypothesis, HKAFs were evaluated on the basis of dose metric distributions generated for a theoretical “younger population.” These distributions were reconstructed by multiplying by 3 the number of individuals of each subpopulation <18 yr, as well as pregnant women, as compared to the numbers that were previously used to reconstruct the Canadian population distributions (Table 3). The number of adults was also adjusted to maintain a total of 100,000 dose metric values. Thus, more than 60% of the resulting “younger population” was aged <18 y, as compared to approximately 20% for the Canadian population.

## 3. Results

### 3.1. Distributions of Internal Dose Metrics in Each Subpopulation and the Entire Canadian Population

**Figure 1:**
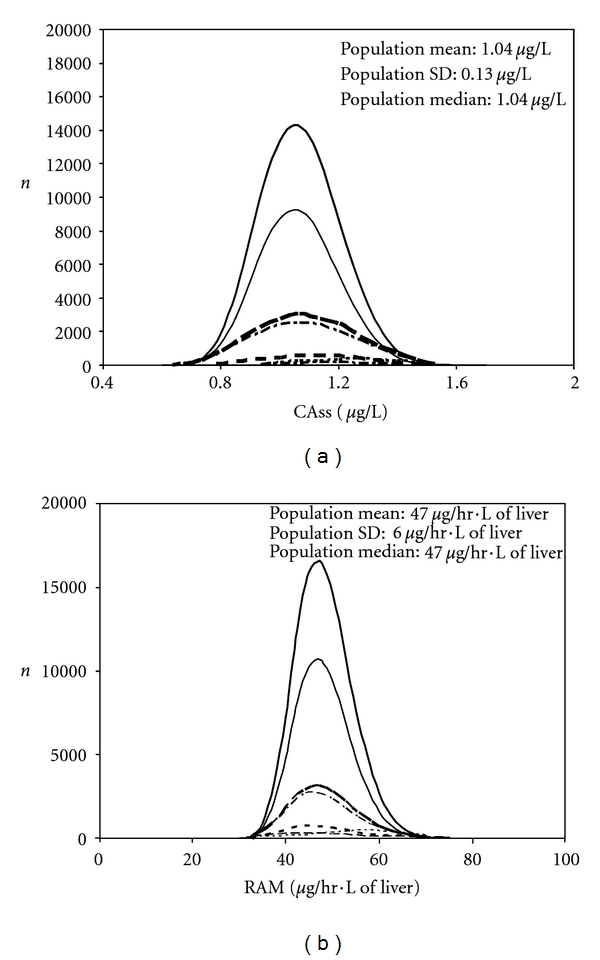
Distributions of individual values obtained for CAss (a) and RAM (b) in each subpopulation within the whole Canadian population for constant inhalation exposure to benzene. From top to bottom, the distributions are shown for the entire Canadian population (thick **—**), adults (—), children and adolescents (**— –**), elderly (–·–·–), toddlers (**– – -**), pregnant women (-------), infants (-·-·-·-), and neonates (indistinguishable).

**Figure 2:**
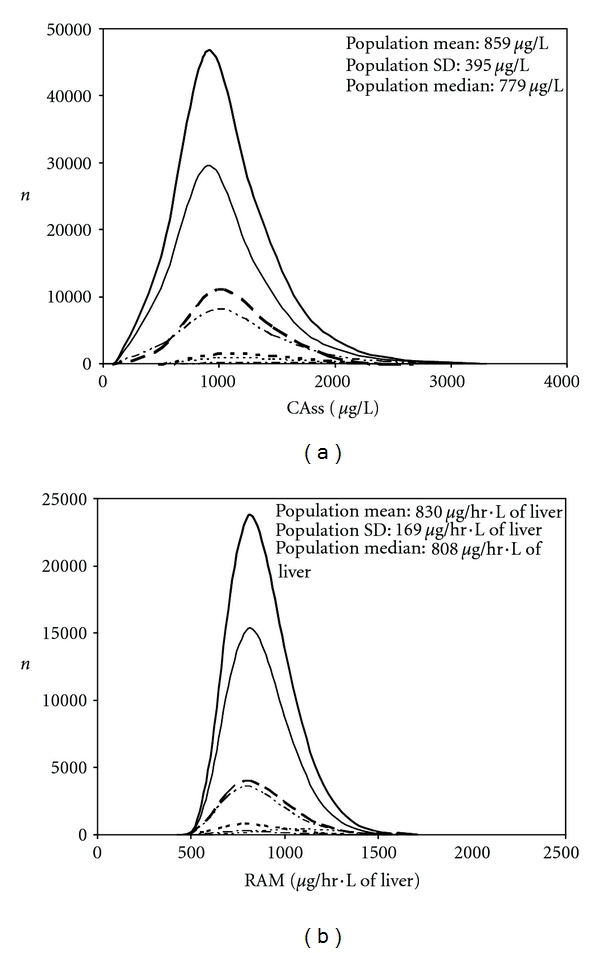
Distributions of individual values obtained for CAss (a) and RAM (b) in each subpopulation within the whole Canadian population for constant inhalation exposure to 1,4-dioxane. From top to bottom, the distributions are shown for the entire Canadian population (thick **—**), adults (—), children and adolescents (**— –**), elderly (–·–·–), toddlers (**– – -**), pregnant women (-------), infants (-·-·-·-), and neonates (indistinguishable).

Figures 1 and 2 show the simulation of internal dose metric distributions in each subpopulation (making up the entire Canadian population) exposed to benzene and 1,4-dioxane, respectively. The shapes of the Canadian population distributions appeared normal for CAss of benzene and lognormal in the other cases. The ranges (1st*–*99th percentile) and median dose metrics that were obtained when simulating 100,000 individuals in each subpopulation are indicated in Table 4. Based on the median and 99th percentile dose metrics, neonates and pregnant women were the most susceptible subpopulations (i.e., they exhibited the highest dose metric) based on CAss and RAM, respectively. The median dose metric in the most susceptible subpopulation was always greater than the median dose metric in the Canadian population, but it was lower than the 99th percentile value, except for the CAss value for 1,4-dioxane. In this case, the median value in neonates (2.3 mg/L) was greater than the 99th percentile value in the whole population (2.14 mg/L). The internal variability of internal dose metrics in the Canadian population can be appraised by the ratio of the 99th to the 1st percentile values. The greatest variability was obtained for 1,4-dioxane based on simulations of CAss exhibiting an approximately sevenfold difference. The population variability was lower in every other case ((99th/1st percentile) ratios lower than 3). Similar trends were obtained for each specific subpopulation, although the magnitude of the differences varied. In particular, neonates exhibited a tenfold (99th/1st percentile) ratio of CAss for 1,4-dioxane. This dose metric exhibits a variability leading to such ratio that is always greater than 5 regardless of the subpopulation. In every other subpopulation and dose metric, the ratio was at most equal to 3 (neonates' RAM for 1,4-dioxane).

### 3.2. HKAF Values

#### 3.2.1. “Whole Population” Approach

HKAFs determined based on the “whole population” approach, which used both the median adult (HKAF_ad_) and the median individual in the entire Canadian population (HKAF_pop_) as referents, are indicated in Table 5. CAss-based HKAFs varied between 1.2 and 1.3 for benzene and between 2.1 and 2.8 for 1,4-dioxane. These values were slightly lower than the highest “distinct subpopulation-” based HKAFs for benzene but were significantly lower than the 1,4-dioxane values (see below). Considering the RAM, all the HKAF values were between 1.2 and 1.6 regardless of the chemical. These values were slightly lower than the highest RAM-based HKAFs obtained with the “distinct subpopulation” approach in pregnant women (1.5*–*2.1, see below). 

#### 3.2.2. “Distinct Subpopulation” Approach

Table 5 shows that the 95th and the 99th percentile-based HKAFs that were computed using the “distinct subpopulation” approach were comparable whether the median adult (HKAF_ad_) or the median individual in the whole population (HKAF_pop_) was used as a referent. In addition to the referent adults, results for neonates and pregnant women are presented because they were, toxicokinetically, the most susceptible based on their respective CAss and RAM (Table 4). HKAFs for infants were also shown because they exceeded the default 3.16 value when CAss of 1,4-dioxane was considered, on the basis of the 99th percentile value (3.8). The default value was also exceeded based only on CAss of 1,4-dioxane in neonates (range: 6.5*–*8.5) and the 99th percentile value in pregnant women (3.5). Neonates exhibited the greatest CAss-based HKAFs for inhaled benzene (1.6*–*1.7). Otherwise, pregnant women showed the greatest RAM-based HKAFs for benzene (1.5*–*1.6) and 1,4-dioxane (1.8*–*2.1). HKAFs in other subpopulations remained in the range of the HKAFs presented in Table 5 for any given dose metric (data not shown).

### 3.3. Proportions of the Whole Population or the Distinct Subpopulations Covered by the Different HKAFs

Table 6 shows the proportion of each subpopulation that was covered by the various HKAFs defined using the “whole population” approach. The 95th or 99th percentile-based “whole population” HKAFs generally protect at least, or very close to, 95% and 99%, respectively, of the individuals of each subpopulation. However, only 57% of the neonates, 78% of the pregnant women, and 89% of the infants were covered by the 95th percentile-based “whole population,” CAss-based HKAF for benzene. Corresponding values for the 99th percentile-based HKAF values were 73%, 92%, and 97%, respectively. In the case of 1,4-dioxane, 27%, 76%, and 86% of the neonates, infants, and pregnant women were covered by the 95th percentile-based HKAF, respectively. Corresponding values for the 99th percentile HKAFs were 48%, 92%, and 96%, respectively, and the default 3.16 factor appears to cover only 60% of the neonates. Considering the RAM, the lack of coverage by the “whole population-” based HKAF concerns pregnant women, as only 63% and 85% of them are covered by, respectively, the 95th and 99th percentile-based HKAF for benzene. These numbers are 66% and 86% in the case of 1,4-dioxane. Finally, when the HKAF was computed with the “distinct subpopulation” approach and the greatest value was retained, more than 99% of the entire Canadian population was covered for every dose metric considered (Table 6). 

### 3.4. Impact of the Demography on the Computed HKAFs

**Figure 3:**
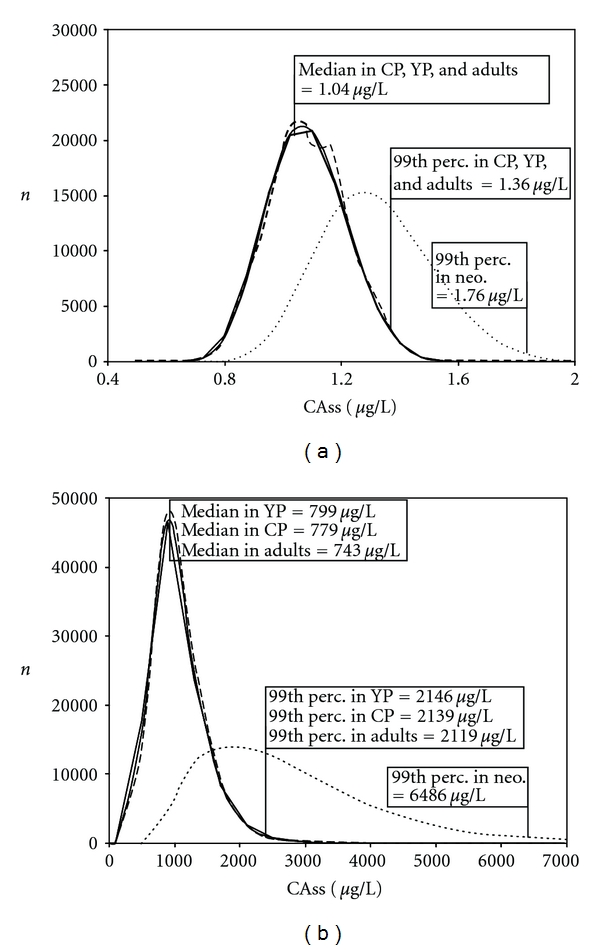
Distribution of individual values obtained for CAss for constant inhalation exposure to benzene (a) and 1,4-dioxane (b) in the entire Canadian (CP, thick **—**) and “younger” (YP, **– –**) populations of 100,000 people, in 100,000 adults (—) and in 100,000 of the most susceptible neonates (neo,…). Median and 99th percentile values only (for clarity reasons) are indicated.

**Figure 4:**
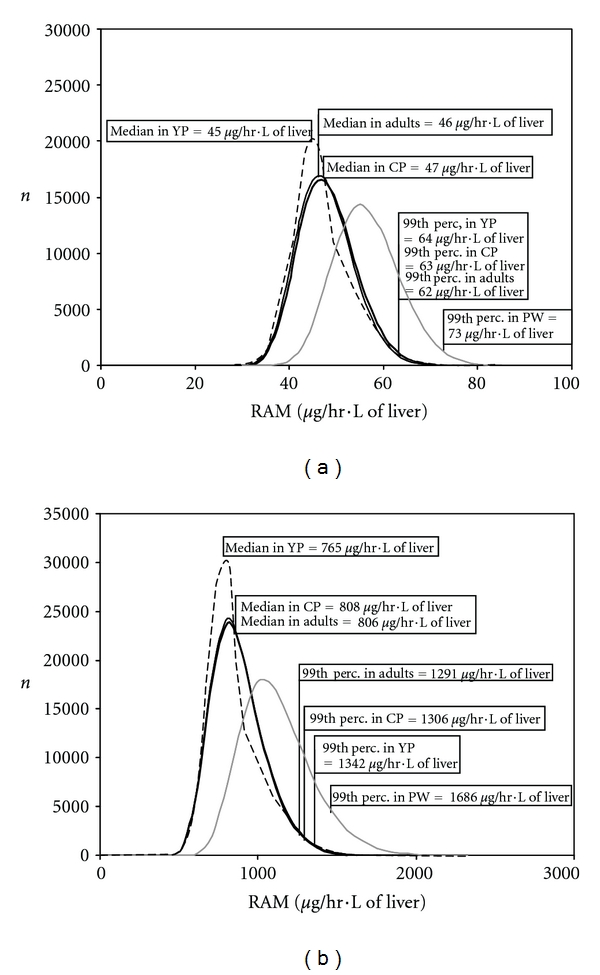
Distribution of individual values obtained for RAM for constant inhalation exposure to benzene (a) and 1,4-dioxane (b) in the entire Canadian (CP, thick **—**), and “younger” (YP, **– –**) populations of 100,000 people, in 100,000 adults (—) and in 100,000 of the most susceptible pregnant women (PW, grey **—**).

The impact of the demographic characteristics on the HKAF values as computed herein can be appreciated from the results shown in Figure 3 for CAss and Figure 4 for RAM. The distributions for 100,000 referents (adult) and the most susceptible individuals (neonates for CAss, pregnant women for RAM) are also shown in these figures for comparison purposes. For benzene (Figure 3(a)), the change in demographics did not impact the overall population distribution of CAss (indistinguishable from adults) and thus, not the HKAF. The change of demographics shifts minimally to the right the population distribution of CAss for 1,4-dioxane (Figure 3(b)). The impact on the various HKAFs was low (“whole population” HKAF = 2.69 versus 2.75) with differences of 3% or less for the relevant statistical descriptors. Considering RAM, when the number of significantly less susceptible neonates, and infants, and more susceptible pregnant women, was increased at the expense of adults, the entire population distribution of the dose metric for both chemicals was widened slightly and particularly for 1,4-dioxane (Figure 4). The impact of pregnant women was apparent with a slight burst in the “younger” population distribution, which was observed near the pregnant women's approximate median value. On the basis of the 99th percentile values, this resulted in virtually unchanged HKAFs for benzene (Figure 4(a)), but marginal changes were observed for 1,4-dioxane (Figure 4(b)). The “whole population” HKAFs that were calculated from the indicated statistical descriptors are 1.67 (1342/806) and 1.75 (1342/765), which were based on median values in adults or population distributions, respectively, for the “younger” population. In comparison, the “whole population” HKAF for the Canadian population was 1.62. The “distinct subpopulation” HKAF for pregnant women was also slightly increased, from 2.09 for the Canadian and adult populations to 2.2 for the “younger” population due to lower median values (765 *μ*g/h-L of liver versus 808 or 806 *μ*g/h-L of liver).

## 4. Discussion

This study performed Monte Carlo simulations on a steady-state algorithm to reconstruct representative subpopulation and whole population distributions of internal dose metrics for continuous inhalation exposure to a highly (benzene) and poorly (1,4-dioxane) cleared chemical. This allowed evaluating the impact of various assumptions on the resulting HKAF.

Virtual populations have been reconstructed to evaluate the population variability of the pharmacokinetic of drugs (e.g., [32, 33]), but to date, the same approach had not been realized for environmental contaminants. This procedure realized in the context of the present study allowed obtaining results showing that the impact of the approach chosen to compute the HKAF depends on the chemical and dose metric considered. The “whole population” approach used here can be related to the “Finite Sample Size” model of IVF from Price et al. [4], whereas the “distinct subpopulation-” based HKAF can be associated to these authors' “sensitive population” model. When the most sensitive individuals, based on dosimetric considerations, constitute a very small fraction of the entire population, a “whole population-” based HKAF might not be sufficient to cover them adequately. For instance, less than 60% of the neonates, constituting less than 0.1% of the whole Canadian population in this study, were covered by the “whole population” HKAF based on their 95th percentile CAss value. This was also the case of infants, who constituted a mere 1% of the entire population, for whom less than 90% of the individuals simulated were covered by that same HKAF (Table 6). The reasons for these results can be determined from Figures 1 and 2. Because toxicokinetically sensitive neonates and infants make up a small proportion of the population, their CAss values do not stand out at the right end of the whole population distributions (Figures 1(a) and 2(a)). Thus, the “distinct subpopulation-” based HKAF would appear to be more adequate in these cases because the focus is then put on the most sensitive subpopulations, regardless of whether the data follow unimodal or bimodal distributions. Conversely, when the more sparse individuals (neonates and infants) are rather less sensitive than the vast majority of the individuals composing the entire population, as for RAM, the approach taken to compute the HKAF does not impact its value (Table 5). 

The results obtained in Figures 3 and 4 can be viewed as a “sensitivity analysis” of the impact of demography on the HKAF. Replacing a significant number of adults from the Canadian population with individuals who are generally equally susceptible as adults (Table 4) resulted in a “younger” population distribution of CAss for benzene that remained virtually unchanged (Figure 3(a)). In the case of 1,4-dioxane (Figure 3(b)), every replacing individual pertained to subpopulations that were more susceptible than adults (Table 4), and, as a result, the population distribution of CAss slightly shifted to the right towards the most susceptible neonates. In the case of RAM, the individuals replacing the adults were either more susceptible (pregnant women) or less susceptible (children), leading to population distributions that were wider for both chemicals (Figure 4). As mentioned in Section 3, the sensitivity of the HKAF to the population demography (i.e., the impact of the population distribution shift on the estimated HKAF) was marginal because the differences in the susceptibilities were not very pronounced between the subpopulations, with the exceptions of neonates and infants based on CAss (particularly for 1,4-dioxane), and pregnant women based on the RAM. However, the impact of these subpopulations' dose metric on the entire population distribution always remained minimal because of their small percentage in the entire population.

**Figure 5:**
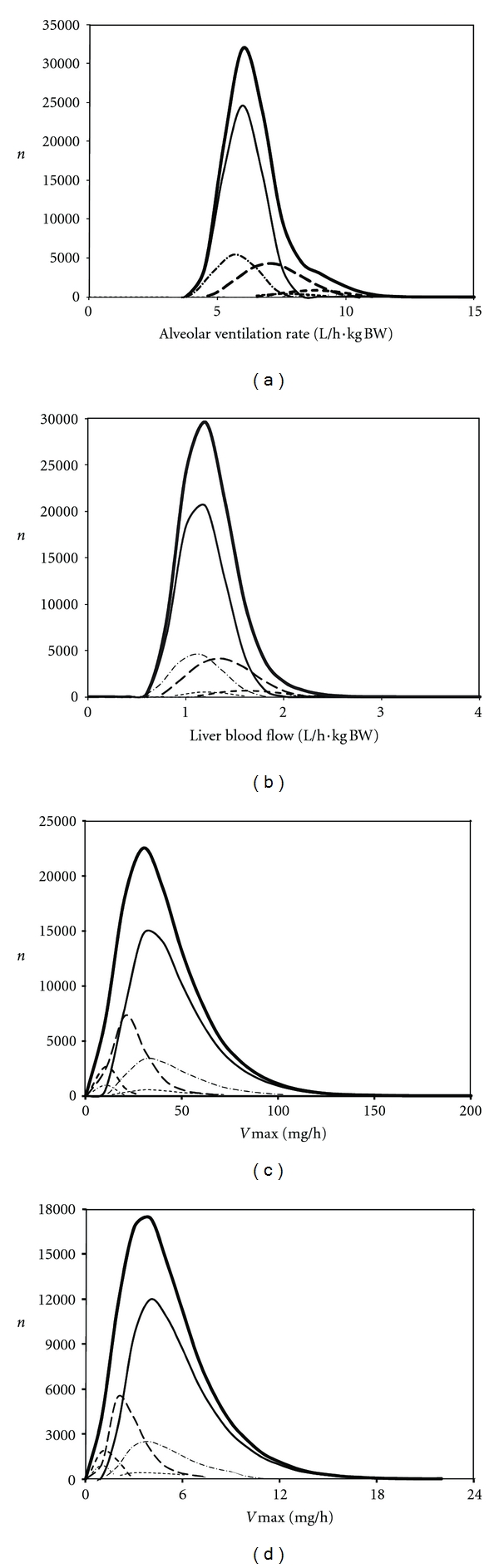
Distributions of individual values obtained for several physiological parameters in each subpopulation within the whole virtual Canadian population. From top to bottom, distributions for body weight-adjusted alveolar ventilation rate (a), body weight-adjusted liver blood flow (b) and maximal rate of metabolism of benzene (c) and 1,4-dioxane (d) are shown for the entire Canadian population ( thick **—**), adults (—), children and adolescents (**— –**), elderly (–·–·–), toddlers (**– – -**), pregnant women (-------), infants (-·-·-·-), and neonates (indistinguishable).

While in our study, demography appears to have, at the most, a very marginal effect on HKAF, the population distributions of CAss are conversely significantly influenced by the determining physiological parameters. Indeed, intake and pulmonary clearance of both benzene and 1,4-dioxane are driven by alveolar ventilation rate, which is rather log normally distributed when adjusted to the body weight (Figure 5(a)). However, blood-flow limited metabolism results in hepatic blood flow being the determining parameter for the clearance of benzene whereas for 1,4-dioxane, hepatic enzyme concentration and thus *V*max⁡ (see (4)) is determinant of its enzyme-limited clearance. As a result, the distribution of body-weight-adjusted liver blood flow (Figure 5(b)), which is more skewed than the distributions of *V*max⁡ (Figure 5(c)) or Clint (central tendency, range: *≈*400 L/h, 0–1600 L/h), yields a population distribution of CAss that is more skewed for benzene (Figure 1(a)) than for 1,4-dioxane (Figure 2(a)). Indeed, the hepatic clearance of the latter is rather driven by the *V*max⁡ (Figure 5(d)) and the corresponding Clint (central tendency, range: *≈*1.7 L/h, 0–7 L/h). Correspondingly, “whole population-” based HKAFs are smaller for benzene than 1,4-dioxane (Table 5).

The toxicokinetic determinants, including physiological parameters, of the susceptibility of each subpopulation to a given chemical based on any dose metric have been thoroughly discussed elsewhere [16]. Briefly, neonates are the most susceptible population based on CAss (Table 5) because they are exposed to a greater-than-adult body-weight-adjusted dose by inhalation, or to a poorly metabolized chemical (1,4-dioxane) for which hepatic metabolism is enzyme-limited, thus reduced in neonates. For pregnant women, their greater susceptibility on the basis of RAM is due to their increased intake on a body weight basis (due to high Qp) combined with their efficient hepatic clearance, a combination that yields a high rate of conversion of inhaled parent compound into metabolites. Greater inhalation uptake on a body weight basis and corresponding blood concentration of inhaled VOCs, for young children and pregnant women as compared to adults, have been consistently documented and discussed in the literature [14–16, 29, 34–39]. The systemic clearance of high *P*_*b*_, poorly metabolized 1,4-dioxane is Qp-dependent (for pulmonary clearance) and enzyme-dependent (for hepatic clearance), and the greater intrasubpopulation variability of CAss that was observed for this chemical was expected. This greater variability results in neonates and infants constituting the only subpopulation for which the consideration of the 99th percentile value rather than the 95th significantly changes their HKAF value (Table 5). Else, the intra-subpopulation variability was rather low for every dose metric. 

The “distinct subpopulation-” based HKAF_ad_ that was obtained for benzene exposure in infants (1.3*–*1.4, Table 5) and toddlers (1.25*–*1.33, not shown) was very similar to the values obtained for other inhaled VOCs by Pelekis et al. [40] for a 10 kg child. Using a deterministic steady-state approach, these authors obtained an average factor of 1.1 ± 0.6 for eight chemicals highly cleared by either pulmonary or hepatic clearance or both. Also, a ratio of the neonate's 95th percentile value to the adult's median value of blood concentrations for dichloromethane was slightly above 2 in the study by Pelekis et al. [41] for continuous inhalation exposure, as compared to 1.6 for benzene in the current study. Besides, the “distinct subpopulation-” based HKAF_pop_ that was obtained in neonates for 1,4-dioxane (6.5, Table 5) was markedly greater than the value (2) obtained by Mörk and Johanson [17] for acetone, a chemical that is similar to 1,4-dioxane (poorly metabolized and highly water soluble with a *P*_*b*_ of 260). The HKAF obtained for 1,4-dioxane in children and adolescents (1.8, not shown) is comparable to those obtained by these authors for 10 and 15 yr old children for acetone (1.7–2.4). The discrepancy for neonates might be explained by the difference in the mean age considered (14 days versus 3 months) and related hepatic enzyme content. The “whole population-” based HKAF_pop_ obtained here based on the 95th percentile (2.1) compares well to Mörk and Johanson's results (i.e., 1.9). Finally, Renwick and Lazarus [7] determined that more than 99% of individuals in a theoretical population of 1 million would be covered by the default factor, a proportion also obtained in this study. 

Among the limitations of this study are other demographic characteristics, including gender differentiation, that could have been considered when generating the population distributions. In particular, ethnicity can be a critical determinant of population variability in toxicokinetics [7] because it is often linked to polymorphic metabolism [42]. However, multiplying subpopulation categories would increase the uncertainty linked to analyzing the distribution of the dose metric in very rare individuals like those with genetic polymorphisms. Besides, gender-related differences in the blood toxicokinetics of several VOCs have been considered insignificant [38, 43]. Furthermore, ethnicity is likely not a primary factor determining CYP2E1 activity because the population variability in the enzyme expression caused by factors other than polymorphism, such as ethanol consumption and xenobiotic coexposure [44] is considerable. Another limitation relates to the use of only healthy individuals in this study; the HKAFs calculated thus do not account for diseased people with altered hepatic or extrahepatic clearance.

In conclusion, this study has, for the first time, systematically compared different approaches for computing the HKAF under various assumptions related to the population/subpopulation variability in internal dose metric for continuous inhalation exposure. This was determined for two environmental chemicals exhibiting different patterns of systemic clearance, to encompass a range of other potential chemicals with such characteristics. This study contributes to clarify the implications of the different underlying assumptions that relate to the interindividual variability considered when determining the HKAF for any risk management consideration, including adequate coverage or not of the most susceptible, but sparse, individuals of a given population. In this regard, relying on the “distinct subpopulation” approach appears more conservative (protective) as it better covers the most susceptible individuals, in particular if they compose a small proportion of the general population. Fundamentally, the difference in the extent of coverage afforded by these two approaches would appear to depend upon the proportion of the most sensitive individuals in the target population for a risk assessment. Moreover, the present work has illustrated the feasibility of a novel approach for characterizing demography-based population variability of internal dose metrics for environmental contaminants.

## Figures and Tables

**Table 1:** Chemical-specific parameters used in the steady-state algorithm.

Parameter	Chemical	
	Benzene^(a)^	1,4-Dioxane^(b)^
*V*max_c_ (mg/h-kg^0.75^)	2.11	0.27
Km (mg/L)	0.1	3.0
Blood : air partition coefficient (*P_b_*)	7.4	3650
Exposure concentration (mg/m^3^, UF × RfC)^(c)^	0.3	3

^(a)^[22].  ^(b)^[23].  ^(c)^[10].

BW: body weight; Km: Michaëlis-Menten constant, *P_b_*: blood : air partition coefficient, RfC: reference concentration; UF: interindividual uncertainty factor; *V*max_c_: constant maximum rate of metabolism.

**Table 2:** Physiological parameters distributions used in the Monte Carlo simulations of the internal dose metrics with the steady-state algorithm.

Parameter^(a)^	Subpopulation						
	Median age (range)						
	Adults 41 (18–64)	Neonate 14 d (0–30 d)	Infants 6.5 m (1–12 mo)	Toddlers 2 (1–3)	Children and adolescents 12 (4–17)	Elderly 78 (65–90)	Pregnant women 29 (15–44)
Sampled parameters							
Body weight (BW) (Kg: m ± SD, range)	76 ± 17, 37–152^(b)^	4 ± 1, 2–7^(c)^	9 ± 2, 3–15^(b)^	13 ± 2, 7–32^(b)^	36 ± 16, 9–113^(b)^	72 ± 16, 33–155^(b)^	70 ± 18, 36–152^(d)^
[CYP2E1] (pmol/mg MSP: m ± SD, range)	49 ± 2, 11–130^(e)^	18 ± 14, 1–56^(c)^	36 ± 21, 10–86^(c)^	42 ± 18, 18–74^(c)^	53 ± 23, 22–95^(c)^	^(f)^	^(f)^
Calculated parameters^(b), (g)^							
Alveolar ventilation rate (Qp)	= [((0.2519 × BW^0.7609^) + (0.2508 × BW^0.7815^))/2)] × *“*variability term” (i.e., 1 ± 0.1 (0.8–1.2))						
Liver volume (Vl)	= 0.05012 × BW^0.78^ × *“*variability term” (i.e., 1 ± 0.14(0.66–1.34))						
Liver blood flow (Ql)	= 0.92 × Vl × *“*variability term” (i.e., 1 ± 0.13 (0.67–1.33))						

^(a)^Log normal distributions for sampled parameters and normal distributions for “variability terms.” All indicated means are arithmetic, except note  ^(e)^(see below).  ^(b)^P^3^M database [28].   ^(c)^[19].  ^(d)^Distribution for non-pregnant women taken from P^3^M database [28], and, to each of these values, the mean body weight increase at any week during pregnancy (normal distribution of 5 ± 4.4 kg (0–14.1)) based on data from ICRP was added [16, 29].  ^(e)^(Geometric mean ± geometric standard deviation) [20].  ^(f)^Same as for adults.  ^(g)^[16].

CYP2E1: cytochrome p-450 2E1; MSP: microsomal protein; SD: standard deviation.

**Table 3:** Reconstruction of the hypothetical populations of 100,000 people with the Canadian demographic profile.

	Canadian population in 2009^(a)^	
Subpopulation (age range)	Median age = 39.7 yr	
	Population size (%)	Corresponding reconstructed population size and number (*n*) of Monte Carlo iterations
Adults (18–64)	21,685,253 (63.92)	63,923
Neonates (0–30 d)	31,303 (0.09)	93
Infants (1–12 mo)	344,329 (1.02)	1,015
Toddlers (1–3)	1,126,896 (3.32)	3,322
Children and adolescents (4–17)	5,382,420 (15.87)	15,866
Elderly (65–90)	4,634,673 (13.66)	13,662
Pregnant women^(b)^ (15–44)	718,950 (2.12)	2,119

TOTAL	33,923,824 (100)	100,000

^(a)^[30].  ^(b)^Based on a pregnancy rate of 104/1,000 in U.S. women aged 15*–*44  yr [31].

**Table 4:** Distribution statistics of various dose metrics in each subpopulation based on 100,000 Monte Carlo iterations and the entire Canadian populations for constant inhalation exposure.

Subpopulation Statistics	Dose metrics			
	Benzene		1,4-Dioxane	
	CAss	RAM	CAss	RAM
Adults				
1st percentile	0.76	35	285	531
Median	1.04	46	763	806
99th percentile	1.36	62	2119	1291
Neonates				
1st percentile	0.88	20	682	371
Median	1.26	39	2299	686
99th percentile	1.76	55	6486	1149
Infants				
1st percentile	0.8	33	420	526
Median	1.11	45	1150	787
99th percentile	1.44	60	2928	1246
Toddlers				
1st percentile	0.79	35	382	534
Median	1.08	47	968	804
99th percentile	1.38	60	2099	1271
Children and adolescents				
1st percentile	0.77	35	352	538
Median	1.04	47	774	814
99th percentile	1.35	61	1744	1293
Elderly				
1st percentile	0.76	35	286	530
Median	1.04	46	766	807
99th percentile	1.37	62	2144	1291
Pregnant Women				
1st percentile	0.85	41	372	673
Median	1.16	55	995	1050
99th percentile	1.49	73	2698	1686
Canadian population				
1st percentile	0.76	35	299	533
Median	1.04	47	779	808
99th percentile	1.36	63	2139	1306

CAss: blood concentration of parent compound (*μ*g/L); RAM: rate of metabolism (*μ*g/h-L of liver).

**Table 5:** HKAFs obtained by the “distinct subpopulation” approach on the basis of 100,000 Monte Carlo iterations in adults, neonates, infants, and pregnant women and by the “whole population” approach for the Canadian population.

HKAF assumption	Dose metrics			
	Benzene		1,4-Dioxane	
	CAss	RAM	CAss	RAM
“Whole population” approach				
HKAF_ad_^(a)^				
Based on 95th percentile	1.2	1.3	2.1	1.4
Based on 99th percentile	1.3	1.4	2.8	1.6
HKAF_pop_^(b)^				
Based on 95th percentile	1.2	1.2	2.1	1.4
Based on 99th percentile	1.3	1.4	2.8	1.6
“Distinct subpopulation” approach				
In adults				
HKAF_ad_^(c)^				
Based on 95th percentile	1.2	1.2	2.1	1.4
Based on 99th percentile	1.3	1.3	2.8	1.6
HKAF_pop_^(d)^				
Based on 95th percentile	1.2	1.2	2.0	1.4
Based on 99th percentile	1.3	1.3	2.7	1.6
In neonates				
HKAF_ad_^(c)^				
Based on 95th percentile	*1.6*	1.1	*6.6*	1.2
Based on 99th percentile	*1.7*	1.2	*8.5*	1.4
HKAF_pop_^(d)^				
Based on 95th percentile	*1.6*	1.1	*6.5*	1.2
Based on 99th percentile	*1.7*	1.2	*8.3*	1.4
In infants				
HKAF_ad_^(c)^				
Based on 95th percentile	1.3	1.2	3.1	1.4
Based on 99th percentile	1.4	1.3	3.8	1.6
HKAF_pop_^(d)^				
Based on 95th percentile	1.3	1.2	3.0	1.4
Based on 99th percentile	1.4	1.3	3.8	1.5
In pregnant women				
HKAF_ad_^(c)^				
Based on 95th percentile	1.4	*1.5*	2.7	*1.8*
Based on 99th percentile	1.5	*1.6*	3.5	*2.1*
HKAF_pop_^(d)^				
Based on 95th percentile	1.4	*1.5*	2.6	*1.8*
Based on 99th percentile	1.5	*1.6*	3.5	*2.1*

Italicized values indicate the highest HKAF among each subpopulation for a given dose metric.

^(a)^Computed as the ratio of the upper percentile value in the Canadian population (95th or 99th) to the median in 100,000 adults.  ^(b)^Computed as the ratio of the upper percentile value in the Canadian population (95th or 99th) to its median.  ^(c)^Computed as the ratio of the upper percentile value in the subpopulation (95th or 99th) to the median in adults.  ^(d)^Computed as the ratio of the upper percentile value in the subpopulation (95th or 99th) to the median in the Canadian population.

CAss: blood concentration of parent compound; HKAF_(ad/pop)_: human kinetic adjustment factor using either the median in adult (“ad”) or whole population (“pop”) as referent; RAM: rate of metabolism.

**Table 6:** Percentage of individuals in the diverse Canadian subpopulations that are covered by the HKAF and the default factor for various dose metrics and chemicals.

Subpopulation Variability descriptor	Dose metrics			
	Benzene		1,4-Dioxane	
	CAss (%)	RAM (%)	CAss (%)	RAM (%)
Adults				
“whole population”^(a)^ HKAF_95th_	96	96	95	97
“whole population”^(a)^ HKAF_99th_	99	>99	>99	>99
Default 3.16 factor	100	100	>99	100
Neonates				
“whole population”^(a)^ HKAF_95th_	57	100	27	100
“whole population”^(a)^ HKAF_99th_	73	100	48	100
Default 3.16 factor	100	100	60	100
Infants				
“whole population”^(a)^ HKAF_95th_	89	97	76	97
“whole population”^(a)^ HKAF_99th_	97	>99	92	>99
Default 3.16 factor	100	100	97	100
Toddlers				
“whole population”^(a)^ HKAF_95th_	93	97	92	96
“whole population”^(a)^ HKAF_99th_	99	>99	99	99
Default 3.16 factor	100	100	>99	100
Children and adolescents				
“whole population”^(a)^ HKAF_95th_	96	96	98	95
“whole population”^(a)^ HKAF_99th_	>99	>99	>99	>99
Default 3.16 factor	100	100	100	100
Elderly				
“whole population”^(a)^ HKAF_95th_	95	96	95	96
“whole population”^(a)^ HKAF_99th_	>99	99	99	>99
Default 3.16 factor	100	100	>99	100
Pregnant Women				
“whole population”^(a)^ HKAF_95th_	78	63	86	66
“whole population”^(a)^ HKAF_99th_	92	85	96	86
Default 3.16 factor	100	100	98	100
Canadian population				
Greatest^(b)^ “distinct subpopulation” HKAF_95th_	>99	>99	>99	>99
Greatest^(b)^ “distinct subpopulation” HKAF_99th_	>99	>99	>99	>99
Default 3.16 factor	100	100	>99	100

^(a)^Based on the median value in the whole Canadian population.  ^(b)^Based on the median value in adults in Table 5.

CAss: blood concentration of parent compound (*μ*g/L); RAM: rate of metabolism (*μ*g/h-L of liver).

## Abbreviations

CSAF: Chemical-specific adjustment factor
CAss: Arterial blood concentration at steady-state
CYP: Cytochrome P-450
HKAF: Human kinetic adjustment factor
HKAF_(ad/pop)_: Human kinetic adjustment factor using as referent the median in either the adults (ad) or in the population (pop)
IVF: Interindividual variability uncertainty factor
Km: Michaelis-Menten constant
*P*_*b*_: Blood : air partition coefficient
PBPK: Physiologically based pharmacokinetic
POD: Point of departure
Ql: Liver blood flow
Qp: Alveolar ventilation rate
RAM: Rate of metabolism
RfC: Reference concentration
RfD: Reference dose
*V*max⁡: Maximum rate of metabolism
Vl: Volume of liver
VOC: Volatile organic compound.
